# Low-Value Prostate-Specific Antigen Test for Prostate Cancer Screening and Subsequent Health Care Utilization and Spending

**DOI:** 10.1001/jamanetworkopen.2022.43449

**Published:** 2022-11-22

**Authors:** David D. Kim, Allan T. Daly, Benjamin C. Koethe, A. Mark Fendrick, Daniel A. Ollendorf, John B. Wong, Peter J. Neumann

**Affiliations:** 1 Center for the Evaluation of Value and Risk in Health (CEVR), Institute for Clinical Research and Health Policy Studies (ICRHPS), Department of Medicine, Tufts Medical Center, Boston, Massachusetts; 2 Tufts University School of Medicine, Boston, Massachusetts; 3 Biostatistics, Epidemiology, and Research Design (BERD) Center, ICRHPS, Department of Medicine, Tufts Medical Center, Boston, Massachusetts; 4 Department of Internal Medicine and Health Management and Policy, University of Michigan, Ann Arbor; 5 Division of Clinical Decision Making, Department of Medicine, Tufts Medical Center, Boston, Massachusetts

## Abstract

**Importance:**

Delivering low-value care can lead to unnecessary follow-up services and associated costs, and such care cascades have not been well examined in common clinical scenarios.

**Objective:**

To evaluate the utilization and costs of care cascades of prostate-specific antigen (PSA) tests for prostate cancer screening, as the routine use of which among asymptomatic men aged 70 years and older is discouraged by multiple guidelines.

**Design, Setting, and Participants:**

This cross-sectional study included men aged 70 years and older without preexisting prostate conditions enrolled in a Medicare Advantage plan during January 2016 to December 2018 with at least 1 outpatient visit. Medical billing claims data from the deidentified OptumLabs Data Warehouse were used. Data analysis was conducted from September 2020 to August 2021.

**Exposures:**

At least 1 claim for low-value PSA tests for prostate cancer screening during the observation period.

**Main Outcomes and Measures:**

Utilization of and spending on low-value PSA cancer screening and associated care cascades and the difference in overall health care utilization and spending among individuals receiving low-value PSA cancer screening vs those who did not, adjusting for observed characteristics using inverse probability of treatment weighting.

**Results:**

Of 995 442 men (mean [SD] age, 78.0 [5.6] years) aged 70 years or older in a Medicare Advantage plan included in this study, 384 058 (38.6%) received a low-value PSA cancer screening. Utilization increased for each subsequent cohort from 2016 to 2018 (49 802 of 168 951 [29.4%] to 134 404 of 349 228 [38.5%] to 199 852 of 477 203 [41.9%]). Among those receiving initial low-value PSA cancer screening, 241 188 of 384 058 (62.8%) received at least 1 follow-up service. Repeated PSA testing was the most common, and 27 268 (7.1%) incurred high-cost follow-up services, such as imaging, radiation therapy, and prostatectomy. Utilization and spending associated with care cascades also increased from 2016 to 2018. For every $1 spent on a low-value PSA cancer screening, an additional $6 was spent on care cascades. Despite avoidable care cascades, individuals who received low-value PSA cancer screening were not associated with increased overall health care utilization and spending during the 1-year follow-up period compared with an unscreened population.

**Conclusions and Relevance:**

In this cross-sectional study, low-value PSA tests for prostate cancer screening remained prevalent among Medicare Advantage plan enrollees and were associated with unnecessary expenditures due to avoidable care cascades. Innovative efforts from clinicians and policy makers, such as payment reforms, to reduce initial low-value care and avoidable care cascades are warranted to decrease harm, enhance equity, and improve health care efficiency.

## Key Points

**Question:**

To what extent is an initial low-value prostate-specific antigen (PSA) test for prostate cancer screening associated with subsequent health services utilization and costs?

**Findings:**

In this cross-sectional study of 995 442 men aged 70 years and older, 38% received a low-value PSA cancer screening between 2016 and 2018, and more than half received follow-up services, with repeated PSA testing being the most common. For every $1 spent on a low-value PSA cancer screening, an additional $6 was spent on follow-up care.

**Meaning:**

In this study, low-value PSA screening for prostate cancer increased from 2016 to 2018 and was associated with unnecessary expenditures due to avoidable care cascades, despite multiple guidelines recommending against its routine use in men aged 70 years and older.

## Teaser

This cross-sectional study evaluates the utilization and costs of care cascades of low-value prostate-specific antigen tests for prostate cancer screening among men aged 70 years and older.

## Introduction

The term *cascading effect* refers to “a process [that], once started, proceeds stepwise to its full, seemingly inevitable, conclusion.”^1^ In the context of medical care, it refers to follow-up testing or treatment after an unclear or abnormal finding from prior service.^2^ Care cascades can occur for various reasons and may include a range of testing and procedures from simple to complex, each involving potential benefits and harms. A survey of practicing US internists indicated that almost all respondents reported experiencing cascading tests or treatments after incidental findings without clinically meaningful beneficial outcomes.^3^

Although care cascades can follow high-value services when the benefit-risk balance favors performing the service, it typically involves more complex or invasive testing that could increase the risk of harm and lead to additional health care use.^4^ Despite the lack of consensus on the meaning of value in health care,^5^ health services that provide little or no benefit, have the potential to cause harm, incur unnecessary costs, or waste limited health care resources, have been considered low-value care,^6,7^ and its prevalent use has been extensively studied.^8,9,10,11,12,13^ A framework for and empirical evidence on downstream consequences from the initial low-value care have also begun to emerge more recently,^14^ including vitamin D testing, preoperative electrocardiograms, and annual wellness visits.^15,16,17^ A recent study found that 62% of the 626 low-value care recommendations from Choosing Wisely (an initiative of more than 80 medical societies and organizations to develop evidence-based guidelines for low-value care) had a high potential for triggering downstream services.^18^

In 2012, updating from the earlier I statement (ie, insufficient evidence) for men aged 70 to 74,^19^ the US Preventive Services Task Force (USPSTF) recommended against PSA screening for prostate cancer, regardless of age (ie, D Grade), because it found that “benefits of PSA-based screening for prostate cancer do not outweigh the harms”^20^ (no further change was made for men aged 70 and older in the 2018 update).^21^ The American Urological Association and the American College of Physicians also do not recommend routine PSA screening in men aged 70 years or older or in any man with less than a 10- to 15-years’ life expectancy.^22,23^ PSA screening for men aged 70 years and older could lead to greater harms from false-positive results for cancers, invasive diagnostic biopsy, and treatment related to overdiagnosis and overtreatment, including more costly procedures, such as biopsy, imaging, and prostatectomy.^24,25,26^ In a previous study, 11% of men who received initial PSA screening needed additional tests to confirm whether they did or did not have cancer.^24^ Despite multiple guidelines recommending against its routine use in this population, low-value PSA tests for prostate cancer screening remain prevalent.^12,27,28,29^ However, to our knowledge, potentially avoidable subsequent utilization and costs of health services associated with the initial low-value PSA cancer screening have not been examined. Our study aimed to investigate care cascades of low-value PSA tests for prostate cancer screening among men 70 years of age and older enrolled in a Medical Advantage plan by analyzing: (1) utilization of and spending on low-value PSA cancer screening and associated care cascades and (2) the difference in overall health care utilization and spending among individuals after a low-value PSA cancer screening vs individuals without one.

## Methods

### Data Source and Population Selection

Our target population consisted of Medicare Advantage plan enrollees from January 1, 2016, to December 31, 2018, in the deidentified OptumLabs Data Warehouse (OLDW) based on medical billing claims for more than 40 million beneficiaries with private insurance. The OLDW data include beneficiaries’ demographic characteristics (including race and ethnicity information), socioeconomic characteristics, medical services received, cost of care, and instances of clinical diagnoses for disease information. Self-reported race and ethnicity groups were Asian, Black, Hispanic, White, and missing. Race and ethnicity was included to estimate the association between the receipt of low-value PSA cancer screening and individual characteristics. This study followed the Strengthening the Reporting of Observational Studies in Epidemiology (STROBE) reporting guideline for cross-sectional studies and was approved by the Tufts Medical Center/Tufts University Health Sciences institutional review board. The requirement for informed consent was waived due to the use of existing deidentified data.

To define a low-value PSA cancer screening, we used the USPSTF’s D grade,^30^ recommending against routine PSA tests for prostate cancer screening in men aged 70 years or older, supported by other clinical guidelines as well.^22,23^ We excluded individuals with poor prostate health (ie, elevated PSA level) and prostate cancer diagnosis within 12 months before the observation, using *International Classification of Diseases, Ninth Revision *(*ICD-9*) and *International Statistical Classification of Diseases and Related Health Problems, Tenth Revision *(*ICD-10*) diagnostic codes from a published algorithm for identifying this population in medical billing claims.^8^ We converted *ICD-9* to *ICD-10* codes using the OptumLabs Crosswalk (*ICD-9*: 185, 233.4, 790.93, V16.42, V76.44, 236.5, V10.46, 600; *ICD-10*: C61, D07.5 D40.0, R97.2, Z80.42). We also required individuals to have 1 year of continuous enrollment from the date of their index event to capture subsequent services within the first several months resulting from a positive PSA screening and treatments.

### Care Cascades of Low-Value PSA Cancer Screening

Among those who received initial low-value PSA cancer screening, we assessed the proportion of individuals who received associated care cascades using the following services: an additional PSA test, a prostate biopsy, imaging of the prostate, radiation of the prostate, or a prostatectomy.^31,32,33^ We used *ICD-10 Procedure Coding System* and *Current Procedural Terminology*/Healthcare Common Procedure Coding System to identify these services (eTable 1 in the Supplement). In addition, we examined the total cost (ie, payer contribution and beneficiary out-of-pocket cost) in the entire population and per 100 000 for these services and estimated the mean unit cost per service. We calculated the mean unit procedure cost by dividing the total amount paid on the claim of service by the total number of people who received this claim.

### Statistical Analysis

#### Likelihood of Receiving Low-Value PSA Cancer Screening

We created PSA and non-PSA groups, requiring both groups to have at least 1 outpatient visit, with a specific claim for a PSA test in the PSA group. To estimate the probability of receiving a PSA cancer screening (ie, propensity score), we used a logistic regression model, controlling for age, race and ethnicity, homeownership, census region, age-adjusted Charlson Comorbidity Index (CCI), and baseline (1-year prior) health care utilization and spending. We examined the association between observed characteristics and the receipt of the low-value PSA cancer screening.

#### Difference-in-Differences Analysis

Using a difference-in-difference (DID) analysis, we further estimated whether the receipt of low-value PSA cancer screening was associated with changes in 1-year health care visits and medical spending before and after the index visit, compared with those who had an outpatient visit without receiving low-value PSA cancer screening. We first applied the inverse probability of treatment weighting (ie, propensity weighting) to adjust for the imbalance in baseline characteristics between the 2 groups. We then estimated health care utilization and spending during the year following the index outpatient visit between the PSA and non-PSA groups after the propensity weighting. Finally, we estimated the differences in incremental changes in 1-year health care utilization and spending between the two groups before and after the index visit:

DID = (Outcome_1-year after PSA_ − Outcome_1-year before PSA_) − (Outcome_1-year after, no PSA_ − Outcome_1-year before, no PSA_).

Total health care utilization involved all visits in any care setting (ie, inpatient, long-term care facility, outpatient, office visits, emergency department, and others) between the initial PSA service date and 365 days later. Overall health care spending included the primary payer contribution and the beneficiary’s out-of-pocket responsibility for all billed medical claims for procedures or services from the date of the index event through 365 days later. We performed the statistical analyses between September 2020 and August 2021 using SAS software version 9.4 (SAS Institute) and Stata Statistical Software release 15 (StataCorp).

## Results

### Sample Characteristics

Our study included 995 442 men aged 70 years or older with at least 1 outpatient visit from 2016 to 2018 (Table 1). Overall, 384 058 (38.6%) received a PSA cancer screening, and utilization increased for each subsequent cohort from 2016 to 2018 (49 802 of 168 951 [29.4%] to 134 404 of 349 228 [38.5%] to 199 852 of 477 203 [41.9%]). The unweighted PSA group, compared with the unweighted non-PSA group, was younger (mean age, 76.63 [95% CI, 76.58-76.68] years vs 78.87 [95% CI, 78.83-78.91]), included more Hispanic participants (28 804 [7.5%] vs 28 735 [4.7%]), was more likely to live in the South (171 290 [44.6%] vs 207 259 [33.9%]), and had a lower mean CCI (1.46 [95% CI, 1.44-1.48] vs 1.87 [1.85-1.88]). The PSA group also had lower baseline health care utilization (mean visits, 22.49 [95% CI, 22.21-22.77] vs 23.93 [95% CI, 23.69-24.17]) and lower mean health care spending ($10 891 [95% CI, $10 651-$11 131] vs $14 515 [95% CI, $14 286-$14 744]) 1 year before the index event. In addition, most of the sample population owned their home (327 601 [85.4%] in the PSA group and 516 008 [84.4%] in the non-PSA group). After inverse probability weighting (ie, propensity weighting) for further analyses, the characteristics of the non-PSA group and PSA group were more balanced (Table 1). The 2018 cohort contributed the most, with 477 203 beneficiaries (PSA group, 199 852; non-PSA group, 277 351); the 2017 cohort had 349 288 (PSA group, 134 404; non-PSA group, 214 884); the 2016 cohort included 168 951 (PSA group, 49 802; non-PSA group,  119 149). Unweighted sample characteristics of each cohort are available in eTable 2 in the Supplement.

**Table 1.** Sample Characteristics of the Non-PSA and PSA Group, 2016 to 2018

Characteristic	Unweighted, No. (%)		After inverse probability weighting, % (95% CI)	
	Non-PSA (n = 611 384)	PSA (n = 384 058)	Non-PSA	PSA
Index year				
2016	119 149 (19.5)	49 802 (13.0)	NA	NA
2017	214 884 (35.1)	134 404 (35.0)	NA	NA
2018	277 351 (45.4)	199 852 (52.0)	NA	NA
Age, mean (95% CI), y	78.87 (78.83-78.91)	76.63 (76.58-76.68)	78.06 (78.05-78.07)	77.97 (77.95-77.99)
Age group, y				
70-74	160 794 (26.3)	156 312 (40.7)	31.8 (31.7-32.0)	31.9 (31.7-32.0)
75-79	175 467 (28.7)	130 196 (33.9)	30.7 (30.6-30.8)	30.7 (30.6-30.9)
80-84	136 339 (22.3)	62 601 (16.3)	20.0 (19.9-20.1)	20.0 (19.9-20.1)
≥85	138 784 (22.7)	34 949 (9.1)	17.5 (17.4-17.5)	17.4 (17.2-17.6)
Race and ethnicity				
Asian	11 616 (1.9)	10 370 (2.7)	2.2 (2.2-2.3)	2.2 (2.2-2.3)
Black	47 077 (7.7)	31 877 (8.3)	8.0 (7.9-8.1)	8.1 (8.0-8.2)
Hispanic	28 735 (4.7)	28 804 (7.5)	5.9 (5.8-6.0)	5.9 (5.8-6.0)
White	454 870 (74.4)	270 761 (70.5)	72.8 (72.6-72.9)	72.7 (72.5-72.8)
Missing	69 086 (11.3)	42 246 (11.0)	11.1 (11.1-11.2)	11.1 (11.0-11.2)
Home ownership				
Own	516 008 (84.4)	327 601 (85.4)	84.8 (84.7-84.9)	84.7 (84.6-84.9)
Does not own	26 290 (4.3)	14 210 (3.7)	4.1 (4.0-4.1)	4.2 (4.1-4.2)
Missing	69 086 (11.3)	42 246 (11.0)	11.1 (11.1-11.2)	11.1 (11.0-11.2)
Census region				
New England	108 215 (17.7)	71 435 (18.6)	18.1 (18.0-18.2)	18.1 (18.0-18.2)
Midwest	233 549 (38.2)	106 768 (27.8)	34.1 (34.0-34.3)	33.9 (33.8-34.1)
South	207 259 (33.9)	171 290 (44.6)	38.0 (37.9-38.2)	38.1 (38.0-38.3)
West	62 361 (10.2)	34 565 (9.0)	9.8 (9.7-9.8)	9.8 (9.7-10.0)
CCI score, mean (95% CI)	1.87 (1.85-1.88)	1.46 (1.44-1.48)	1.71 (1.71-1.72)	1.74 (1.73-1.75)
During 1-y prior to the index event				
Health care visits, No.				
Mean (95% CI)	23.93 (23.69-24.17)	22.49 (22.21-22.77)	23.61 (23.54-23.69)	23.32 (23.22-23.42)
Median, No.	15	15	15	16
Medical spending, $				
Mean (95% CI)	14 515 (14 286-14 744)	10 891 (10 651-11 131)	13 534 (13 455-13 614)	12 349 (12 247-12 451)
Median, $	3862	3377	3646	3641
During 1-y after the index event				
Health care visits, No.				
Mean (95% CI)	33.13 (32.89-33.37)	30.04 (29.75-30.32)	32.46 (32.37-32.55)	31.14 (31.03-31.25)
Median, No.	23	22	22	22
Medical spending$				
Mean (95% CI)	20 580 (20 351-20 809)	16 835 (16 595-17 075)	19 824 (19 725-19 923)	18 330 (18 199-18 461)
Median, $	6818	5989	6530	6400

Abbreviations: CCI, Charlson comorbidity index; NA, not applicable; PSA, prostate-specific antigen.

### Care Cascade of the Initial Low-Value PSA Cancer Screening

**Figure 1.**
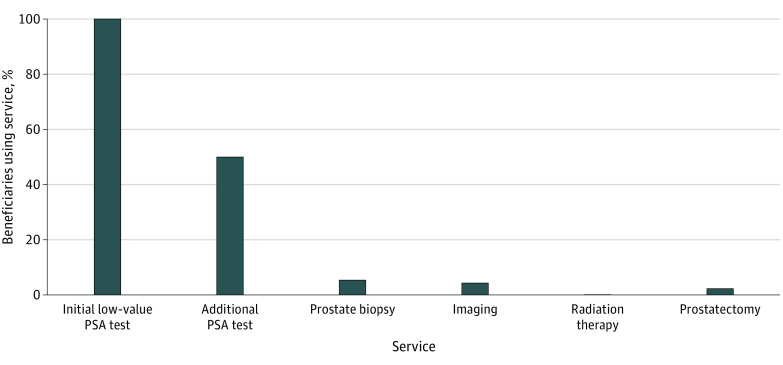
Cascading Care of Low-Value Prostate-Specific Antigen (PSA) Cancer Screening in a Medicare Advantage Population, 2016 to 2018

**Figure 2.**
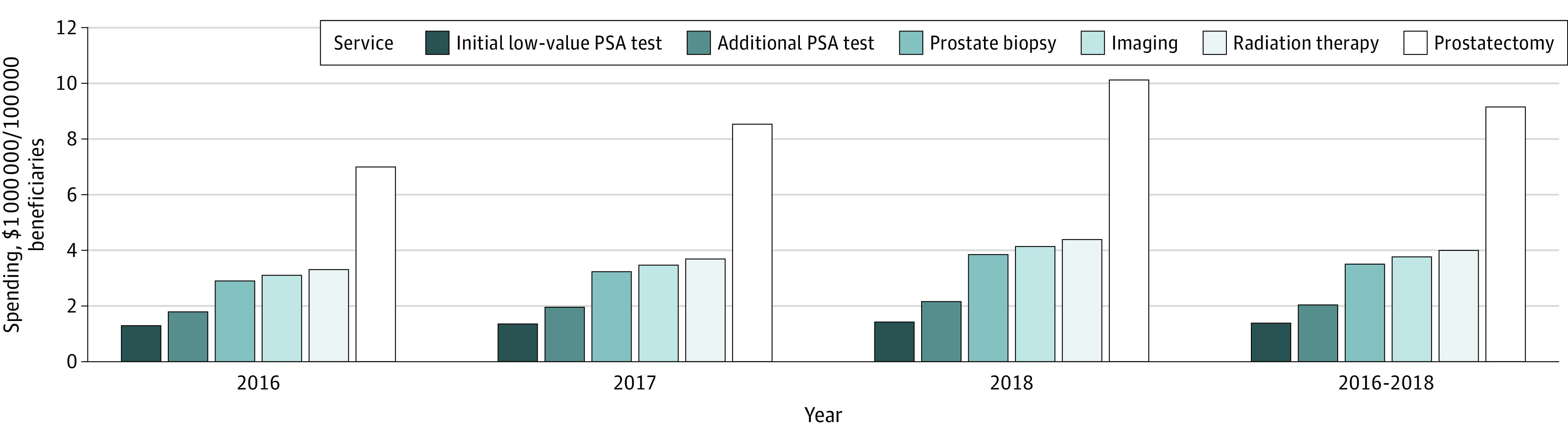
Associated Spending of Cascading Care of Low-Value Prostate-Specific Antigen (PSA) Cancer Screening in a Medicare Advantage Population, 2016 to 2018

We found that the use of and expenditures for follow-up services increased from 2016 to 2018. Among the 384 058 individuals who received a low-value PSA cancer screening, the percentage of those who received at least 1 follow-up service expanded from 49.1% (24 453 of 49 802) in 2016 and 58.3% (78 358 of 134 404) in 2017 to 69.4% in 2018 (138 697 of 199 852), for a total of 62.8% of patients (241 188) receiving at least 1 follow-up service in 2016 to 2018. Overall, the most common follow-up service was additional PSA testing (192 413 [50.1%]), followed by prostate biopsy (21 123 [5.5%]), imaging (17 283 [4.5%]), prostatectomy (9217 [2.4%]), and prostate radiation (768 [0.2%]) (Figure 1). For every $1 spent on a low-value PSA test for prostate cancer screening, an additional $6 was spent on care cascades. (Figure 2) The mean unit procedure cost of the initial PSA cancer screening was $14 (that of subsequent PSA tests was $13), and prostatectomy was the most expensive ($2093) of the follow-up services, followed by radiation ($1204), prostate biopsy ($268), and imaging ($56). Overall, 27 268 patients (7.1%) incurred high-cost follow-up services. Total spending on initial PSA cancer screening for our sample population was $3.8 million, with an additional $22.0 million spent on follow-up services: $1.8 million on additional (ie, second or more) PSA tests, $4.2 million on prostate biopsy, $0.7 million for imaging services, $0.6 million on radiation therapy, and $14.7 million on prostatectomy. Per 100 000 beneficiaries who received low-value PSA cancer screening, $9 million was spent on the initial testing and follow-up services. eTable 3 in the Supplement presents year-specific cascading care of low-value PSA cancer screening and associated spending from 2016 to 2018.

### Likelihood of Receiving Low-Value PSA Cancer Screening

Our logistic regression model found an increased likelihood of receiving low-value PSA tests for prostate cancer screening in populations who were younger, Hispanic, or Asian; owned a home; had fewer baseline comorbidities; had higher baseline health utilization; and reported lower baseline health care costs (Table 2). For example, those aged 85 years or older had an odds ratio of 0.28 (95% CI, 0.28-0.29) for having low-value PSA cancer screening vs those aged 70 to 74 years. Similarly, when considering baseline health care costs in quintiles, the highest quintile had an odds ratio of 0.60 (95% CI, 0.59-0.61) vs those in the lowest quintile. When considering baseline visit utilization, those in the highest quintile were 1.98 (95% CI, 1.94-2.02) times more likely to have low-value PSA cancer screening vs those in the lowest quintile. Year-specific analyses showed similar findings (eTable 4 in the Supplement).

**Table 2.** Logistic Regression Model Results for Having a Low-Value PSA Screening, 2016 to 2018

Characteristic	Odds ratio (95% CI)
Age, y	
70-74	1 [Reference]
75-79	0.79 (0.78-0.80)
80-84	0.49 (0.49-0.50)
≥85	0.28 (0.28-0.29)
Race and ethnicity	
Asian	1.41 (1.37-1.45)
Black	1.01 (0.99-1.02)
Hispanic	1.56 (1.53-1.59)
White	1 [Reference]
Missing	0.96 (0.94-0.97)
Home ownership	
Own	1 [Reference]
Does not own	0.79 (0.77-0.80)
Missing	Omitted
Census region	
New England	1 [Reference]
Midwest	0.68 (0.67-0.68)
South	1.21 (1.20-1.23)
West	0.80 (0.79-0.81)
Charlton Comorbidity Index	0.90 (0.90-0.91)
Total health care visits, quintile	
First, lowest	1 [Reference]
Second	1.20 (1.18-1.21)
Third	1.46 (1.43-1.48)
Fourth	1.71 (1.68-1.74)
Fifth, highest	1.98 (1.94-2.02)
Total medical spending (scaled), quintile	
First, lowest	1 [Reference]
Second	0.87 (0.86-0.88)
Third	0.82 (0.80-0.83)
Fourth	0.76 (0.74-0.77)
Fifth, highest	0.60 (0.59-0.61)
Index year	1.00 (1.00-1.00)

Abbreviation: PSA, prostate-specific antigen.

### Association Between Initial Low-Value PSA Cancer Screening and Changes in 1-Year Health Care Visits and Health Care Spending

**Figure 3.**
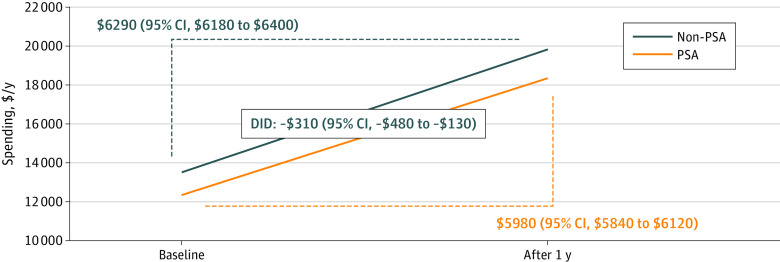
Difference-in-Differences (DID) Analysis of Total Health Care Spending Between the Prostate-Specific Antigen (PSA) Screening Group and Non-PSA Screening Group After Inverse Probability Weighting Those who received the initial low-value PSA cancer screening were associated with increased health care spending by $5980 (95% CI, $5840-$6120) during the 1 year after the PSA screening (mean, $18 330) relative to baseline health care spending during 1-year prior to the PSA screening (mean, $12 350). Those who did not receive PSA screening were associated with increased health care spending by $6290 (95% CI, $6180-$6400). The final DID estimates found that, after accounting for changes from the baseline, the PSA group, on average, spent $310 ($130-$480) less than the non-PSA group.

Although the models estimated increasing visits compared with the prior year’s utilization for both groups (7.8 [95% CI, 7.7 to 7.9] additional visits for the PSA group vs 8.9 [95% CI, 8.9 to 9.0] additional visits for the non-PSA group), the PSA group had 1 fewer visit than the non-PSA group (DID estimate, −1.03 [95% CIs, −1.14 to −0.91] visits) (eFigure 1 in the Supplement). Similarly, the models estimated increased annual health care spending, compared with the prior year in both groups ($5980 [95% CI, $5840 to $6120] for the PSA group vs $6290 [95% CI, $6184 to $6396]) for the non-PSA group), but, on average, the PSA group incurred $310 (95% CI, $130 to $480) less than the non-PSA group (Figure 3).

## Discussion

Our study found that, despite multiple guidelines recommending against its routine use in men aged 70 years and older, PSA tests for prostate cancer screening were highly prevalent among Medicare Advantage beneficiaries and associated with substantial amounts of potentially avoidable care. Total spending associated with care cascades increased from 2016 to 2018, despite relatively unchanged mean unit costs per service, suggesting that the increased use of the low-value PSA cancer screening exacerbated the avoidable wasteful spending. For every $1 spent on a low-value PSA cancer screening, an additional $6 was spent on the associated care cascade. A previous study based on 2007 to 2009 Medicare fee-for-service data^34^ reached a similar conclusion that downstream procedures accounted for 72% of the overall cost of PSA-based prostate cancer screening. When extrapolating our findings to the 1.8 million annual low-value PSA cancer screening costing $46.5 million in the traditional Medicare population,^35^ the care cascades of low-value PSA cancer screening alone could amount to more than $275 million. These estimates of wasteful spending are likely conservative because we only captured procedure costs, excluding additional costs for the office visit, facility, and physician fees for surgical procedures.

Despite avoidable care cascades, the initial low-value PSA tests for prostate cancer screening were not associated with increased overall health care utilization and spending during the 1-year follow-up period compared with an unscreened population. This may be due to the small proportion ultimately receiving expensive therapy for prostate cancer, such as radiation therapy or prostatectomy (2.6% of those having an initial low-value PSA cancer screening and 1.0% of our overall Medicare Advantage sample). In addition, although we applied inverse probability of treatment weighting to adjust for observed differences between these groups, unmeasured potential confounding characteristics, such as the severity of comorbidities and patient preferences, were not completely captured in our statistical adjustment.

Our study also found that individuals who were younger, self-reporting as Hispanic or Asian, and homeowners; had fewer comorbidities; were living in the South; had higher baseline health utilization; and reported lower baseline health care costs were more likely to receive low-value PSA tests for prostate cancer screening. Although our study was not focused on examining potential drivers of receiving a low-value service, further research is needed to better understand the role of patient preferences, geographical variation, and racial-ethnic differences in receiving low-value care services.^36,37,38^

Outside of low-value PSA cancer screening, other studies have documented care cascades of low-value services. A recent Canadian study found that 3 low-value preventive services (ie, chest radiograph, electrocardiogram, and Papanicolaou test) increased the likelihood of having more subsequent visits related to the initial low-value service.^39^ Other studies highlighted the infrequent, yet costly, care cascade of low-value electrocardiograms, urinalyses, and thyrotropin tests in a fee-for-service Medicare population.^15,17^ Considering the clinical nuance of various low-value services, the related care cascades might be service-specific and not be generalizable to different types of services across disease areas, clinicians, or local practice patterns.

### Limitations

Our study has limitations. First, we relied on the multiple guidelines’ recommendations to define a low-value PSA test for prostate cancer screening among men aged 70 years or older. Nevertheless, some PSA screening and downstream services in this population could be deemed necessary and not be considered low-value. Claims data do not provide sufficient clinical context and nuance to determine the necessity of the services provided, such as symptoms individuals are experiencing, results from testing, and why clinicians ordered particular services. Further research is warranted to better assess whether the downstream costs are associated with any clinical benefit, such as the supplementary use of electronic health records data to identify patients with high-risk prostate cancer (Gleason score ≥7). Also, our study sample is limited to a privately insured Medicare population. Although PSA cancer screening rates in our sample were similar to those reported in other studies that included beneficiaries enrolled in either traditional Medicare or other Medicare Advantage plans or those who were dual-eligible,^35^ the overall spending estimates would likely be different among traditional Medicare and Medicare Advantage populations. Additionally, we may have underestimated wasteful spending associated with care cascades of low-value PSA cancer screening by excluding other costs beyond the procedure costs, such as the office visit, facility, and physician fees for surgical procedures. Also, the rapidly increasing costs of imaging in recent years (eg, attributed to the use of multiparametric magnetic resonance imaging [mpMRI] for evaluating elevated PSAs and more expensive fusion biopsies using mpMRI images) are not captured in our data from 2016 to 2018, which could be another reason for underestimation.

## Conclusions

Despite multiple guidelines recommending against its routine use in men age 70 years or older, a third of men in this group received a low-value PSA test for prostate cancer screening. More than half of those screened received subsequent follow-up services, mostly repeated PSA testing, leading to potential harm and additional unnecessary expenditures. For every $1 spent on a low-value PSA cancer screening, an additional $6 was spent on the subsequent care cascade. Because guideline recommendations alone might not lead to long-term sustained effects of reducing low-value PSA cancer screening,^40^ innovative and perhaps harsher efforts to reduce both initial unneeded care and avoidable cascading effects—such as the implementation of Section 4105 of the Patient Protection and Affordable Care Act, which provides the Secretary of Health and Human Services the authority to provide no payment for USPSTF grade D services—may be warranted to decrease harm, enhance equity, and improve efficiency of medical spending.

## Notes

Supplement. **eTable 1.** Identifying Follow-up Care Directly Attributable to the Initial PSA Test **eTable 2.** Study Characteristics of the Non-PSA and PSA Group: 2016, 2017, and 2018 (Unweighted) **eTable 3. **Annual Utilization and Spending of Select Follow-up Services From a Low-Value PSA Screening: 2016, 2017, and 2018 **eTable 4. **Logistic Regression Model Results for Having a Low-Value PSA Screening: 2016, 2017, and 2018
