# Supplementary material for: Low-Value Prostate-Specific Antigen Test for Prostate Cancer Screening and Subsequent Health Care Utilization and Spending
Source: JAMA Netw Open. 2022 Nov 22;5(11):e2243449. doi: 10.1001/jamanetworkopen.2022.43449 (PMC9682424; doi:10.1001/jamanetworkopen.2022.43449)
Supplement: Supplement. — eTable 1. Identifying Follow-up Care Directly Attributable to the Initial PSA Test eTable 2. Study Characteristics of the Non-PSA and PSA Group: 2016, 2017, and 2018 (Unweighted) eTable 3. Annual Utilization and Spending of Select Follow-up Services From a Low-Value PSA Screening: 2016, 2017, and 2018 eTable 4. Logistic Regression Model Results for Having a Low-Value PSA Screening: 2016, 2017, and 2018 [file jamanetwopen-e2243449-s001.pdf]

Supplemental Online Content

Kim DD, Daly AT, Koethe BC, et al. Low-value prostate-specific antigen test for prostate cancer screening and subsequent health care utilization and spending. *JAMA Netw Open.* 2022;5(11):e2243449. doi:10.1001/jamanetworkopen.2022.43449

eTable 1. Identifying Follow-up Care Directly Attributable to the Initial PSA Test

eTable 2. Study Characteristics of the Non-PSA and PSA Group: 2016, 2017, and 2018 (Unweighted)

eTable 3. Annual Utilization and Spending of Select Follow-up Services From a Low-Value PSA Screening: 2016, 2017, and 2018

eTable 4. Logistic Regression Model Results for Having a Low-Value PSA Screening: 2016, 2017, and 2018

This supplemental material has been provided by the authors to give readers additional information about their work.

© 2022 Kim DD et al. *JAMA Network Open.*

eTable 1. Identifying Follow-up Care Directly Attributable to the Initial PSA Test

CPT/HCPCS for specific PSA test-related services

Service	Code	CE Category
Biopsy	55700, 55705, 55706, G0416	Biopsy
Brachytherapy, Needle Insertion, Interstitial Radioelement Application	55875	Radiation
Destruction	55873	Prostatectomy
Thermotherapy	53850, 53852, 0421T, 0582T, 53854	Radiation
Excision	55801, 55821, 55831, 55801, 55810, 55812, 55815, 55831, 55866, 55840, 55842, 55845, 55866, 55821, 52402, 52601, 52630, 0443T 52450	Prostatectomy
Resection	52601, 52630	Prostatectomy
Ultrasound	76872, 76873	Imaging
Prostatectomy	52601, 55801, 55810, 55812, 55815, 55831, 55840, 55842, 55845, 55866, 55821, 52601, 52630	Prostatectomy
PSA	G0103, 84152, 84153, 84154	PSA

© 2022 Kim DD et al. *JAMA Network Open.*

ICD-10 PCS for specific PSA test-related services

Service	All codes that start with	Specific Codes	CE Category
Destruction of Prostate	0V5	0V500ZZ<br>0V503ZZ<br>0V504ZZ<br>0V507ZZ<br>0V508ZZ	Prostatectomy
Excision of Prostate	0VB	0VB00ZX<br>0VB00ZZ<br>0VB03ZX<br>0VB03ZZ<br>0VB04ZX<br>0VB04ZZ<br>0VB07ZX<br>0VB07ZZ<br>0VB08ZX<br>0VB08ZZ	Prostatectomy
Radiation	0VH	0VH001Z<br>0VH031Z<br>0VH041Z<br>0VH071Z<br>0VH081Z<br>0VH403Z<br>0VH40YZ<br>0VH433Z<br>0VH43YZ<br>0VH443Z<br>0VH44YZ<br>0VH473Z<br>0VH47YZ<br>0VH483Z<br>0VH48YZ	Radiation
Resection	0VT	0VT00ZZ<br>0VT04ZZ<br>0VT07ZZ<br>0VT08ZZ	Prostatectomy
Plain Radiography	BV0	BV030ZZ<br>BV031ZZ<br>BV03YZZ	Imaging

© 2022 Kim DD et al. *JAMA Network Open.*

CT Scan	BV2	BV2300Z<br>BV230ZZ<br>BV2310Z<br>BV231ZZ<br>BV23Y0Z<br>BV23YZZ<br>BV23ZZZ	Imaging
MRI	BV3	BV33Y0Z<br>BV33YZZ<br>BV33ZZZ	Imaging
Ultrasound	BV4	BV49ZZZ	Imaging
Beam Radiation Using Photons	DV0	DV000ZZ<br>DV001ZZ<br>DV002ZZ<br>DV003Z0<br>DV003ZZ<br>DV004ZZ<br>DV005ZZ<br>DV006ZZ	Radiation
HDR Brachytherapy	DV1	DV1097Z<br>DV1098Z<br>DV1099Z<br>DV109BZ<br>DV109CZ<br>DV109YZ<br>DV10B6Z<br>DV10B7Z<br>DV10B8Z<br>DV10B9Z<br>DV10BB1<br>DV10BBZ<br>DV10BCZ<br>DV10BYZ	Radiation
Stereotactic Other Photon Radio Surgery	DV2	DV20DZZ<br>DV20HZZ<br>DV20JZZ	Radiation
Contact Radiation	DVY	DVY07ZZ<br>DVY08ZZ<br>DVY0CZZ<br>DVY0FZZ<br>DVY0KZZ	Radiation
Destruction using waterjet	XV5	XV508A4	Prostatectomy

© 2022 Kim DD et al. *JAMA Network Open.*

Removal	0VP	0VP400Z<br>0VP401Z<br>0VP403Z<br>0VP407Z<br>0VP40JZ<br>0VP40KZ<br>0VP40YZ<br>0VP430Z<br>0VP431Z<br>0VP433Z<br>0VP437Z<br>0VP43JZ<br>0VP43KZ<br>0VP43YZ<br>0VP440Z<br>0VP441Z<br>0VP443Z<br>0VP447Z<br>0VP44JZ<br>0VP44KZ<br>0VP44YZ<br>0VP470Z<br>0VP471Z<br>0VP473Z<br>0VP477Z<br>0VP47JZ<br>0VP47KZ<br>0VP47YZ<br>0VP480Z<br>0VP481Z<br>0VP483Z<br>0VP487Z<br>0VP48JZ<br>0VP48KZ<br>0VP48YZ<br>0VP4X0Z<br>0VP4X1Z<br>0VP4X3Z	Prostatectomy
Extirpation	0VC	0VC00ZZ<br>0VC03ZZ<br>0VC04ZZ<br>0VC07ZZ<br>0VC08ZZ	Prostatectomy

© 2022 Kim DD et al. *JAMA Network Open.*

eTable 2. Study Characteristics of the Non-PSA and PSA Group: 2016, 2017, and 2018 (Unweighted)

	2016		2017		2018	
	Non-PSA N= 119,149 71%	PSA N= 49,802 29%	Non-PSA N= 214,884 62%	PSA N= 134,404 38%	Non-PSA N= 277,351 58%	PSA N= 199,852 42%
**Mean Age**	78.7	76.6	79.0	77.0	79.0	77.0
**Age group**						
70-74	30%	48%	30%	47%	29%	46%
75-79	25%	28%	25%	29%	25%	29%
80-84	20%	14%	20%	14%	20%	14%
85+	25%	11%	25%	10%	26%	11%
**Race/Ethnicity**						
Asian	2%	3%	2%	3%	2%	3%
Black	7%	8%	8%	9%	8%	8%
Hispanic	4%	6%	5%	8%	5%	8%
White	78%	75%	74%	72%	73%	69%
Missing	10%	8%	11%	9%	13%	13%
**Home Ownership**						
Own	86%	87%	85%	87%	83%	84%
Does not own	4%	4%	5%	4%	4%	3%
Missing	10%	8%	11%	9%	13%	13%
**Census Region**						
New England	18%	22%	17%	18%	18%	18%
Midwest	44%	33%	39%	29%	35%	26%
South	27%	36%	34%	44%	37%	47%
West	10%	9%	10%	9%	10%	9%
**Charlton Comorbidity Index**	2.0	1.6	1.9	1.5	1.8	1.4
**Mean total number of healthcare visits**	22	21	24	22	25	23
Median	14	14	15	15	16	16
**Mean total of medical spending**	$9,209	$8,109	$13,985	$10,435	$16,930	$11,729
Median	$2,693	$2,488	$3,811	$3,146	$4,520	$3,751

© 2022 Kim DD et al. *JAMA Network Open.*

eTable 3. Annual Utilization and Spending of Select Follow-up Services From a Low-Value PSA Screening: 2016, 2017, and 2018

	2016<br>N= 49,802	2017<br>N= 134,404	2018<br>N= 199,852	All<br>N=384,058
**Utilization**				
**Initial low-value PSA test**	100%	100%	100%	100%
**At least one follow-up service**	49.1%	58.3%	69.4%	62.8%
Additional PSA Test	38.6%	46.7%	55.4%	50.1%
Prostate Biopsy	4.5%	4.8%	6.2%	5.5%
Imagining of the Prostate	3.8%	4.2%	4.9%	4.5%
Radiation Therapy	0.2%	0.2%	0.2%	0.2%
Prostatectomy	2.0%	2.4%	2.6%	2.4%
**Mean unit cost of care**				
**Initial low-value PSA test**	$13	$14	$14	$14
**All services received in follow-up care**	$66	$80	$94	$85
Additional PSA Test	$13	$13	$13	$13
Prostate Biopsy	$251	$268	$273	$268
Imagining of the Prostate	$51	$56	$57	$56
Radiation Therapy	$1,053	$1,077	$1,328	$1,204
Prostatectomy	$1,848	$2,021	$2,205	$2,093
**Total Spending (per 100,000 beneficiaries)**				
**Initial low-value PSA test**	$1,299,000	$1,357,000	$1,430,000	$1,386,975
**All follow-up care**	$5,710,690	$7,180,379	$8,693,504	$7,765,701
Additional PSA Test	$483,658	$597,293	$721,862	$646,470
Prostate Biopsy	$1,128,240	$1,285,104	$1,695,018	$1,476,145
Imagining of the Prostate	$193,002	$233,142	$278,320	$251,122
Radiation Therapy	$210,670	$215,424	$265,512	$240,707
Prostatectomy	$3,695,120	$4,849,416	$5,732,792	$5,151,256

© 2022 Kim DD et al. *JAMA Network Open.*

eTable 4. Logistic Regression Model Results for Having a Low-Value PSA Screening: 2016, 2017, and 2018

	2016		2017		2018	
	Odds Ratio	95% CI	Odds Ratio	95% CI	Odds Ratio	95% CI
**Age group**						
70-74	Ref		Ref		Ref	
75-79	0.70	(0.69, 0.72)	0.76	(0.75, 0.77)	0.78	(0.77, 0.79)
80-84	0.44	(0.43, 0.46)	0.47	(0.46, 0.48)	0.48	(0.47, 0.49)
85+	0.28	(0.27, 0.29)	0.28	(0.27, 0.29)	0.28	(0.28, 0.29)
**Race and Ethnicity**						
White	Ref		Ref			
Black	1.06	(1.02, 1.11)	0.99	(0.96, 1.02)	1.00	(0.98, 1.03)
Hispanic	1.38	(1.31, 1.45)	1.57	(1.52, 1.62)	1.61	(1.56, 1.65)
Asian	1.38	(1.28, 1.48)	1.45	(1.38, 1.53)	1.43	(1.37, 1.49)
Missing	0.84	(0.81, 0.88)	0.89	(0.87, 0.92)	0.99	(0.97, 1.01)
**Home Ownership**						
Own	Ref		Ref		Ref	
Does not own	0.83	(0.79, 0.88)	0.80	(0.77, 0.83)	0.78	(0.76, 0.81)
**Census Region**						
New England	Ref		Ref		Ref	
Midwest	0.57	(0.55, 0.58)	0.72	(0.71, 0.74)	0.74	(0.73, 0.75)
South	1.00	(0.97, 1.03)	1.25	(1.23, 1.28)	1.27	(1.25, 1.29)
West	0.65	(0.62, 0.68)	0.83	(0.81, 0.85)	0.87	(0.85, 0.90)
**Charlton Comorbidity Index**	0.93	(0.92, 0.94)	0.92	(0.92, 0.93)	0.90	(0.89, 0.90)
**Total healthcare utilization quintiles**						
First	Ref		Ref		Ref	
Second	1.06	(1.02, 1.10)	1.24	(1.21, 1.28)	1.27	(1.24, 1.30)

© 2022 Kim DD et al. *JAMA Network Open.*

Third	1.23	(1.17, 1.29)	1.53	(1.49, 1.58)	1.56	(1.52, 1.60)
Fourth	1.35	(1.28, 1.41)	1.82	(1.76, 1.88)	1.91	(1.85, 1.96)
Fifth	1.40	(1.33, 1.48)	2.06	(1.99, 2.13)	2.34	(2.27, 2.42)
**Total medical spending quintiles**						
First	Ref		Ref		Ref	
Second	0.79	(0.76, 0.82)	0.80	(0.78, 0.83)	0.91	(0.89, 0.93)
Third	0.78	(0.75, 0.82)	0.71	(0.69, 0.74)	0.83	(0.81, 0.86)
Fourth	0.78	(0.74, 0.82)	0.66	(0.63, 0.68)	0.72	(0.70, 0.74)
Fifth	0.69	(0.66, 0.73)	0.52	(0.50, 0.54)	0.54	(0.53, 0.56)

© 2022 Kim DD et al. *JAMA Network Open.*
